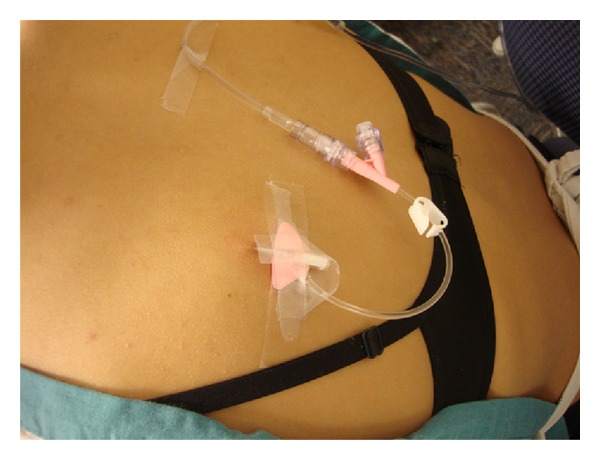# Subcutaneous Fluid Administration: A Potentially Useful Tool in Prehospital Care

**DOI:** 10.1155/2012/904521

**Published:** 2012-05-09

**Authors:** Annette O. Arthur, Jeffrey M. Goodloe, Stephen H. Thomas

**Affiliations:** Department of Emergency Medicine, School of Community Medicine, The university of Oklahoma-Tulsa, Tulsa, OK 74135, USA

## Abstract

Mass casualty incidents (MCIs) and disaster medical situations are ideal settings in which there is need for a novel approach to infusing fluids and medications into a patient's intravascular space. An attractive new approach would avoid the potentially time-consuming needlestick and venous cannulation requiring a trained practitioner. In multiple-patient situations, trained practitioners are not always available in sufficient numbers to enable timely placement of intravenous catheters. The novel approach for intravascular space infusion, described in this paper involves the preadministration of the enzyme, human recombinant hyaluronidase (HRH), into the subcutaneous (SC) space, via an indwelling catheter. The enzyme “loosens” the SC space effectively enhancing the absorption of fluids and medication.

## 1. Introduction

Hyaluronidase-facilitated subcutaneous infusion (HRH-SCI), also denoted by “enzymatically assisted subcutaneous infusion” (EASI), appears based upon available evidence to be an easy, safe, and effective means of infusing fluids and some medications into the intravascular space. The relevance of EASI to the prehospital setting appears to be highest in the disaster/MCI arena, where there may be limited numbers of practitioners treating numerous persons. In short, EASI may be a partial solution to the problem of mismatch between numbers of patients requiring intravascular access and capabilities of available providers to provide that access.

Building on reports of EASI use in other settings, namely, in hospice care and in pediatric patients, prehospital investigators have assessed EASI as placed by first EMT-paramedics (EMT-Ps) [1] and then in a follow-up study with EASI placed by EMT-basic (EMT-B) level providers [2]. In its first two studies in simulated prehospital situations, EASI access was placed with 100% effectiveness and no significant complications.

This paper is not intended to be a comprehensive treatment of the complex subject of prehospital vascular access and fluids/medication administration. Rather, the intent is to briefly outline the possible role for a novel approach to the long-standing challenge of access. The paper will open by making a case for the need to investigate new avenues for intravascular access. Next, we will outline the existing work with EASI access and infusion as performed by prehospital personnel. The paper concludes with consideration of potential future directions for EASI research and application.

## 2. The Rationale for Considering a Novel Route of Access to the Intravascular Space

### 2.1. Importance of Intravascular Access

The most vital component of the case for importance of access to the intravascular space is the consistent message in the literature, that out-of-hospital hydration is often critical. Experts aver that administration of fluids in the out-of-hospital setting remains an important intervention in disaster and MCI circumstances [3–5]. Clinical practice as well as standard emergency medicine and trauma resuscitation teaching emphasizes the importance of early fluid resuscitation for a variety of injuries and illnesses [5]. The literature addressing MCI situations (e.g., crush injuries) makes a strong case for the importance of fluid replacement [3]. Therefore, for fluid resuscitation alone, there is a strong case to be made that access to the intravascular compartment is an important priority for prehospital providers (particularly in disaster/MCI situations). Fluid administration as considered herein includes glucose-containing fluids such as D_5_NS (normal saline with dextrose 5%).

### 2.2. Challenges in Obtaining Vascular Access in the Out-of-Hospital Setting

Despite the widespread acknowledgment of the importance of prehospital intravascular access, obtaining that access through the traditional intravenous (IV) line is not always simple. As is the case with many other procedures, that which is usually straightforward in the hospital and emergency department (ED) setting can be difficult in the field.

The out-of-hospital setting can pose special challenges to the provider attempting to gain access to the intravascular compartment. These challenges can be categorized as being related to establishing an individual patient's IV, or those being related to establishing access in a large number of patients.

In an individual patient encounter, placement of an IV catheter may be hampered by a number of situations. The anatomy may be difficult, as is the case with venous collapse in hypotension. Positioning issues (e.g., entrapment) may render it difficult to reach the vein. IV access placement problems may be compounded by environmental conditions ranging from inadequate lighting to jarring vehicular motion.

In daily practice in the single-patient setting, experienced field practitioners usually rely on training and experience as they overcome IV access difficulties and achieve access. Nonetheless, there are certainly cases in which the problems are not surmounted and prehospital IV access either fails or requires multiple attempts. In these situations, there may be room in the prehospital armamentarium, for a new approach to the intravascular compartment.

The situation with respect to multiple-patient encounters (such as MCI situations) may be characterized by just the same problems as those described for individual attainment of prehospital IV access, but on a larger scale. A few-minute delay in attaining IV access in an individual patient is not often life threatening. That same delay can cause major morbidity in a situation in which a small group of providers is faced with starting dozens of IVs in a difficult situation. In a disaster or MCI situation, there may actually be insufficient healthcare provider resources to place IV access in a timely fashion, even if the IV placement goes smoothly in each individual patient. A lesser concern, but one warranting mention, is the fact that repeated venipuncture attempts may cause pain that is rated as “significant” by patients and parents [6–11].

The “bottom line” of the challenges that can face prehospital providers attempting to establish IV access in the field is that in some cases there is room for a new approach. Whether the problem is repeated IV “sticks” in a given patient, or the need to establish dozens of intravascular access lines in a short time period, there is potential utility in a rapid, easily taught, and simple system for establishing reliable access to the intravascular compartment.

### 2.3. Limitations in Alternatives to IV Access

The need for assessing alternative mechanisms for intravascular compartment access has already been recognized. Noting the significance of the problems that can occasionally be encountered in the field execution of IV access, trauma researchers have investigated alternative methods of hydration, such as proctoclysis, infusion of fluids into the rectum [12]. If the need for alternative methods of fluid administration is sufficient to prompt serious consideration of proctoclysis—the drawbacks of which are not limited to impracticality, the search for alternative routes to the intravascular compartment should include SC infusion (which is much easier to place and better tolerated).

 The main alternative at this time, to IV access, is the intraosseous (IO) line. Just as IV cannulation is the usual answer to the question of how to obtain intravascular access, IO access has historically been the (only) alternative approach. To be clear, there is a definite role for IO lines in out-of-hospital care. However, the myriad situations that may be encountered in the field, combined with the disadvantages of IO lines, make a case for assessing viability of a non-IO approach. IO lines have an acknowledged role in prehospital medicine, but their risks include pain, extravasation, infection, and fracture. IO placement is not necessarily quick, and the capability to perform the procedure requires initial and ongoing skills maintenance [13, 14]. One study found that while 18 of 19 IO lines appeared to be correctly placed on initial attempt, there were 5 cases with serious complications (e.g., dislodgment, extravasation, failure of infusate to flow), leading to an overall IO success rate of 68% [14]. In about a third of the cases, IO placement required over a minute to establish [14].

The just-cited literature does not mean that there is not a role for IO lines in out-of-hospital care. IO is occasionally useful (and even life saving). However, the limitations of IO and other currently used alternatives to IV access do leave room for assessment of a possible role for a new mechanism to get to the intravascular space.

## 3. Existing Evidence and Method for Use of Subcutaneous Infusion

### 3.1. Physiology of HRH-Assisted SC Infusion

A now-outdated term for SC fluid infusion, “hypodermoclysis,” was in use for decades [15]. For all practical purposes, the practice ceased in the USA with the arrival of modern IV catheter equipment, but SC infusion is still in use in some settings [15, 16]. In addition to the fact that “new” (nonmetal) IV catheters were simple to use and quite safe, hypodermoclysis abandonment was due in part to the physiology of the SC interstitial matrix which serves as the main barrier to diffusion of SC-administered fluid.

The extraadipocyte portion of the SC compartment is a gel-like matrix of collagenous fibrils and glycosaminoglycans. Subcutaneously administered drugs and infusates must traverse this interstitial matrix to enter the vascular or lymphatic system. The interstitium resistance to drug permeation can be envisioned as functioning like a three-dimensional filter through which drugs/infusates must pass. There are large molecules such as elastin and collagen inhabiting a matrix of hydrated gel-like glycosaminoglycans and proteoglycans. Among the most important glycosaminoglycans (for purposes of this review) is hyaluronan, which contributes to the resistance of fluid flow through the interstitium. Though hyaluronan is found in lower concentration than collagen in the skin, it plays a disproportionately large role in resisting fluid movement [17].

Hyaluronidase modifies connective tissue permeability via hydrolyzing hyaluronic acid, effecting a cleavage of the glucosaminidic bond between *N*-acetylglucosamine and glucuronic acid moieties. The cleavage results in a decrease in viscosity of the cellular cement and promotes diffusion of injected fluids, facilitating their absorption. The decrease in viscosity is reversed within 24 hours, due to the rapid inactivation of the hyaluronidase enzyme and also due to the rapid turnover rate of skin hyaluronan [18–20].

Hyaluronidase-spreading agents, historically derived from animal extracts, have been used clinically to facilitate dispersion and absorption of other drugs for over 50 years [18]. This long history of successful use contributed to a relatively rapid FDA approval for HRH (in 2005), which states the drug is “indicated as an adjuvant to increase the absorption and dispersion of other injected drugs, for hypodermoclysis, and as an adjunct in subcutaneous urography for improving resorption of radiopaque agents” [21].

### 3.2. EASI Access to the Intravascular Compartment

The HRH used in the most recent literature on SC infusion has as its main advantage over previous hyaluronidase products the absence of immunogenicity. As a human recombinant agent, HRH poses far less allergy/anaphylaxis risk (essentially, none) as compared to animal-derived hyaluronidase. The HRH otherwise works as described above, in facilitating “spread” of fluids and medications administered into the SC space.

The concept of using HRH in actual patients has been demonstrated feasible and effective. The initial clinical experience concentrated on the hospital and hospice settings [18, 22]. Hospice patients needed indwelling catheters for delivery of fluids and analgesia, and pediatric patients in the hospital benefited from rapidly placed lines that served as a mechanism for hydration [23]. These studies revealed that the HRH-facilitated hydration was very easily placed, lasted for many hours (or days) without complication, and alleviated the need for IVs.

The studies with the most likely relevance to the prehospital and MCI situation are probably the EASI Access I and EASI Access II trials (conducted in part by some of this review's authors) [1, 2]. These studies are important, because of two major “new” angles: (1) institution of HRH-facilitated infusion lines by prehospital providers and (2) use of stable-isotope labeling techniques to definitively demonstrate rapid and significant uptake into the intravascular compartment of SC-infused (glucose-containing) fluid.

In EASI Access I, 4 EMT-Ps instituted EASI access in 20 healthy volunteers. The EASI access lines were successfully placed in under 15 seconds, in all study subjects. Normal saline with 5% dextrose (some of which was ^13^C-labeled) was infused at rates that were held at no more than 150 mL/hour. Gas chromatography/mass spectrophotometry (GC/MS) was used to demonstrate uptake of the isotopic glucose in the infusate, as early as 15 minutes into the infusion (this was the first time of analysis). Since the EASI access lines were placed more quickly than IV lines, and since the infusates were administered with zero significant adverse effects, the study concluded that EMT-P administration of fluids and glucose via EASI was likely a viable option for some prehospital circumstances.

EASI Access II followed up the result of EASI Access I, by assessing the placement of HRH-facilitated SC infusion lines by EMT-Bs. This was undertaken after the EASI Access I investigators observed that the placement of the EASI access lines appeared to require very little medical expertise and minimal training. EASI Access II main findings were (1) confirmation of rapid and consistent uptake of SC-infused labeled glucose (as detected with GC/MS at the first assessment time, 5 minutes into infusion) and (2) demonstration of 100% success rates in rapid and effective establishment of EASI access in 18 subjects by 18 EMT-B practitioners (the same 18 individuals served as both study subjects and the EASI line placement operators). In EASI Access II, the infusate of 5% dextrose in water (D_5_W) was administered at a median rate of 400 mL/hour.

### 3.3. Mechanism for Use of EASI Access

 One reason that EASI access appears to be attractive for use in the prehospital setting is that the technique requires minimal training and little in the way of additional supplies. The EASI Access I and II studies each entailed no more than 5–10-minute training (time varied depending on time required by each subject to reach comfort level). Furthermore, the HRH used comes in unit-dose vials that are easily reconstituted before being administered through an initially placed SC line in a dose of 150 units (1 mL). 

**Figure 1:**
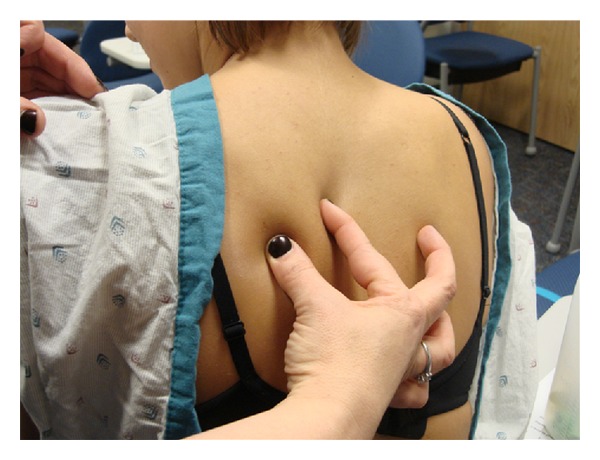


**Figure 2:**
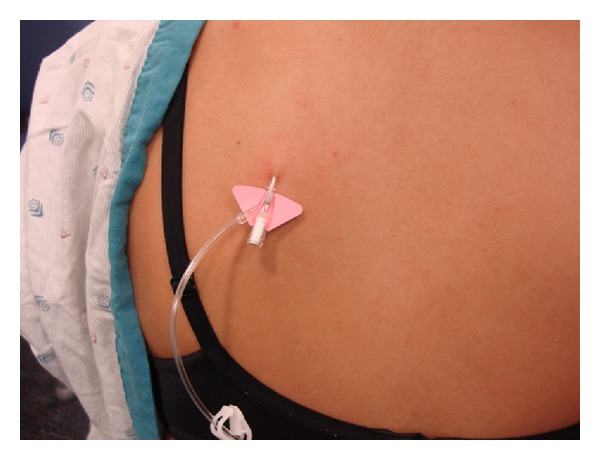


 First comes placement of the SC line. A fold of skin—preferably on the upper back as this area is accessible and allows for painless fluid administration—is pinched (Figure 1). Into the fold is placed a standard IV catheter; 22- or 24-gauge catheter works well but sizes can be varied (Figure 2). After the catheter is taped into place, HRH is administered into the catheter in a painless step that begins by thinning of the SC tissues. Finally, standard IV tubing is to be connected and secured. A photo of an EASI line in place is depicted in Figure 3.

 It is noteworthy that the upper back location is not required. Any location that is accessible in a given situation may serve as the access point for an EASI line. Locations in which the subcutaneous space may be “tight” (e.g., dorsum of the hand) may theoretically be associated with infusion discomfort that is not seen with locations in the upper arm or the back.

Regardless of where EASI is placed, it is sensible to begin with a low infusion rate, increasing the fluid infusion pace only after confirmation of painless infusion at lower rates. The authors have found that infusion rates in the 400 mL/hr range are easily attainable (and painless) [1, 2]. Other investigators have used small-gauge catheters (24 g) and infused isotonic solution (lactated Ringer's) with no discomfort, at maximal gravity-assisted rates exceeding 500 mL/hr [24].

The best fluid to be chosen is probably the one that contains glucose. Glucose is an important component of fluids in prehospital (especially MCI) situations, and EASI Access I and II clearly demonstrated rapid and significant uptake of EASI-infused glucose into the intravascular space [1, 2]. Palliative care and hospice data include reports of painless administration of a breadth of fluids including normal saline (NS) and variants with and without dextrose (e.g., D_5_NS, D_5_ 0.5 NS); 5% dextrose in water (D_5_W) has also been infused without problems [25].

## 4. Future Research and Practice Directions

The preceding information paints a tantalizing, if incomplete, picture of a novel method of fluid administration. Since IV access is markedly more of a problem in the prehospital setting than in the hospital, and since the difficulties are magnified in MCI situations, the relevance of EASI access to out-of-hospital care may prove to be high.

One question that arises is whether prehospital providers can adjudicate which cases may be appropriate for SC infusion. Just as prehospital providers currently exercise (protocol-guided) judgment as to who requires IV (or IO) access, those providers can appropriately triage patients to SC infusion. It appears possible that, in some cases where IV access fails, SC may be a more viable option for rapid fluid and medication administration than IO (e.g., when patient requirements are for minimal fluids and rapid analgesia). The guidance for prehospital personnel and respondents to disasters or MCIs will need to include consideration of both individual patients and aggregate situations.

The existing literature demonstrates administration of a variety of fluids, as well as glucose and opioid analgesia (in the palliative care setting) [15, 24, 25]. Future focus should include assessment of which agents may be administered via EASI, with safety and efficacy. Fluids and analgesia are a reasonable starting point (especially since the latter may be given both SC and IV already), but there remain possibilities for other agents' administration (e.g., midazolam) after appropriate research.

In our area, meteorological disasters (especially tornadoes) occur with unfortunate frequency. Situations in which dozens (or scores) of patients with mild-moderate injury, who need fluid resuscitation, are easily imagined. Our next EASI Access III study is planned to include administration of EASI access in actual prehospital patients for whom IV access fails and in whom prehospital providers do not judge IO placement as appropriate. This study will be a good assessment of the use of EASI access in routine pre-hospital fluid administration. HRH is also being planned for utilization with our local disaster response teams, in anticipation of caring for large numbers of patients or individual disaster patients with need (e.g., entrapment and no IV access). Another use of EASI access worthy of investigation is its effect on subsequent IV access attempts in patients who need improved intravascular volume or with increased interstitial edema.

It appears that the existing evidence, while not establishing a concrete role for EASI access in the prehospital setting, does support the continued investigation of this FDA-approved approach for fluid resuscitation in the field. Time will tell whether SC infusion proves a useful part of the prehospital and disaster armamentarium.

## Figures and Tables

**Figure 3:**